# Thermacut bur gingivectomy versus functional crown lengthening in management of subgingival interproximal carious lesions 12 months randomized controlled trial

**DOI:** 10.1038/s41598-025-99313-0

**Published:** 2025-05-08

**Authors:** Ahmed Khairy Elmorsy, Shereen Hafez Ibrahim, Hani Mohamed Essam El-Nahass, Nesma Shemais, Ahmed El Zohairy

**Affiliations:** 1 Faculty of Dentistry, Cairo University, Cairo, Egypt; 2 Lecturer of Periodontology, Faculty of Dentistry, Cairo University, Cairo, Egypt

**Keywords:** Deep marginal acquisition, Thermacut Bur, Deep subgingival interproximal carious lesions, Functional crown lengthening, Deep caries, Randomized controlled trials, Gingival aesthetics, Bonded restorations

## Abstract

This clinical trial aimed to evaluate acquiring deep subgingival interproximal carious lesions by the mean of Thermacut bur gingivectomy, versus functional crown lengthening procedure. A total of 30 deep proximal cavities in posterior teeth were randomly assigned into two groups (*n* = 15); Thermacut Bur Gingivectomy (TBG) or Functional Crown Lengthening (FCL). Patient Satisfaction using a Visual Analog Scale (VAS) as a primary outcome. Bleeding on Probing (BoP), Probing Depth (PD), and Crestal Bone Level (CBL) as secondary outcomes, and Marginal Integrity using Modified USPHS Criteria as a tertiary outcome, were evaluated at the baseline, immediate postoperative, 6-month, and 1-year follow-up intervals. There was a statistically significant difference between the two groups regarding patient satisfaction in the immediate postoperative values, no statistically significant difference was found in the 6-month and 12-month intervals. Regarding BoP, no statistical significance was found between intervention and control groups at different time intervals. Regarding PD, Pre- and after 6 months, there was no significant difference in values measured in both groups, after 1 year, values measured in the intervention group were statistically significant, Regarding CBL, there was no significant difference in bone levels measured in both groups at baseline. However, in other intervals, values measured in the control group were significantly higher than those in the intervention group. Regarding Marginal Integrity, no significant differences were found between both groups at any time interval. Both Thermacut bur gingivectomy and functional crown lengthening techniques are valid treatments for the management of deep subgingival interproximal carious lesions in vital teeth. However, Thermacut bur was superior to functional crown lengthening in terms of preserving the alveolar bone crest. **Clinical Significance**: Thermacut bur gingivectomy is an alternative to solve the shortcomings of functional crown lengthening.

**Supplementary Information:**

The online version contains supplementary material available at 10.1038/s41598-025-99313-0.

## Introduction

Dental restorations play a critical role in maintaining periodontal health, which is critical for the long-term success of interproximal dental restorations^1^. Deep subgingival interproximal carious lesions can create great challenges during restorative procedures using composite restorations, exposing cavity margins is critical to achieve proper rubber dam isolation, matrix adaptation, adhesion procedure, and composite placement^2^. Minimally invasive dentistry is now becoming the forefront of restorative dentistry, involving less traumatic treatment protocols, and conservation of tooth structure and surrounding tissues, also enhancing the long-term survivability of treated teeth, and improving the overall quality of life for patients. Bringing such philosophy into the clinical workflow can sometimes be challenging as some cases can challenge restorative dentists such as dealing with deep subgingival interproximal carious lesions. Functional crown lengthening was considered the gold standard in managing such cases.

The biologic width is considered an important tight seal around the tooth, which is critical in protecting the periodontium from any microbial injury and maintaining periodontal health^3^. However - in many clinical scenarios - subgingival carious lesions and crown-root fractures may affect the biologic width dimension. Functional crown lengthening is a surgical procedure that is used to restore this important landmark, which plays an important role in maintaining periodontal health and the long-term stability of restorations. Functional crown lengthening is an effective procedure done before the prosthetic procedure with great success, provided that a certain protocol is followed. It is done by achieving at least a 3 mm distance between the alveolar bone crest and the flap margin at the time of suturing^4^.

Several protocols have been proposed to solve this conundrum including the functional crown lengthening, which is considered to be the gold standard of care regarding the management of deep subgingival margins, however, it has its limitations including the risk of causing root exposure, furcation involvement in posterior teeth with high furcation, compromising crown-root ratio, besides risking implant threads exposure if it was performed besides implant, extending crestal bone recontouring to buccal and lingual walls, and in some cases to adjacent teeth in order to attain smooth bony architecture, and the complications of surgery such as postoperative pain, inflammation, edema and the risk of excessive bleeding^5^.

Deep marginal acquisition and deep marginal elevation (DMA & DME) were introduced as a new protocol for managing deep subgingival margins, by using a circumferential matrix to acquire deep margins under rubber dam isolation^2,6^. Other techniques to acquire deep margins have been suggested, like the use of diode laser, electrosurgery, and soft tissue bur^7^. However, there is a new protocol for exposing deep subgingival margins using a thermacut bur, which is a bur with no abrasives to ensure the cutting of the papilla and exposing the margin without the risk of damaging tooth surfaces. It was originally fabricated to remove excess gutta percha after obturation without removing from pericervical dentin area.

Upon reviewing literature for this novel technique using thermacut bur was only mentioned in one research by Venuti P. and Mirabella Eclano., 2018^7^and was described as soft tissue bur as a suggestion for deep margin acquisition and no further studies were found to be related to this interesting and minimally invasive clinical approach. However, this method is currently used by many clinicians with good follow-ups, although it hasn’t been tested in research before. Thus, Thermacut bur gingivectomy (TBG) was chosen as an intervention because it is a novel technique, with no previous studies to test its effect in terms of gingival and periodontal health. Which lead to a profound knowledge gap to be tackled. Functional crown lengthening (FCL) was chosen as the treatment of choice for the control group because it is the gold standard procedure for managing deep interproximal carious lesions^4^.

With limited evidence highlighted, the research question was “Can deep marginal acquisition by means of thermacut bur and deep marginal extension with direct composite restoration be validated in dealing with deep subgingival margins versus functional crown lengthening?” So, this study was designed to compare the ability of two techniques; Deep marginal acquisition by the mean of thermacut bur and functional crown lengthening in dealing with deep subgingival interproximal carious lesions, in terms of patient satisfaction through pain evaluation, Bleeding on Probing, Pocket Depth, Crestal Bone Level evaluation, and restoration evaluation using modified USPHS criteria, to test the null hypothesis that there is no difference between deep marginal acquisition using thermacut bur compared to functional crown lengthening in managing deep subgingival interproximal carious lesions.

## Materials and methods

The materials used in this study were 3 M Filtek one bulk-fill composite (3 M ESPE, Germany), Capo Flowable Bulkfill Composite (Shuetz Dental), Uni-Etch tooth conditioner gel (Bisco, USA) and All bond universal adhesive (Bisco, USA). All the Materials’ specifications, composition, LOT numbers, and manufacturers are presented in Table (1).

**Table 1:** Materials & Armamentarium used with details.

**Material**	**Specifications**	**Composition**	**Manufacturer**	**LOT#**
*Uni-Etch *	A surface-conditioning agent used for enamel and/or dentin treatment prior to adhesive application.	Uni-Etch tooth conditioner gel consists of water, 32% phosphoric acid, Benzalkonium Chloride (BAC), silicon dioxide, surfactants and blue colorant.	Bisco, USA	E-5502EBM
*All bond universal adhesive*	One component universal adhesive that can be used with. It is characterized by compatible with ALL cements, no postoperative sensitivity.	All bond universal adhesive consists of 10-MDP, ethanol, BIS-GMA, HEMA, water, initiator and stabilizer	Bisco, USA	2400000216
*Filtek one Bulkfill Posterior composite*	A radiopaque, light curable composite restorative material used for posterior restorations.	AUDMA, UDMA and 1, 12-dodecane-DMA Composite Filler: zirconia/silica cluster filler (comprised of 20 nm silica and 4 to 11 nm zirconia particles) and ytterbium trifluoride filler consisting of agglomerate 100 nm particles. Filler loading: 76.5% wt. % (58.4% vol.)	3M, Germany	053M4863 A2
*Capo Flowable Bulkfill* *Composite*	a light-curing posterior composite resin for the direct filling therapy and for restorations usingBulk Filling. It is suitable for layering with a thickness of up to 4 mm..	Glass powder, aliphatic urethane dimethacrylate, tetramethylene di-methacrylate, silicon dioxide Total filler: 77 % by weight (57 % by volume) inorganic filler (0.005–40 μm)	Shuetz Dental	2022001873

### Study settings

The protocol of this study was registered on clinical trials (www.clinicaltrials.gov) with I.D.: NTC06205459 (16/01/2024). This randomized controlled clinical trial followed the Consolidated Standards of Reporting Trials (CONSORT) Statement. This study was approved by the Ethics in Human Research Committee of the Faculty of Dentistry, Cairo University with the Identification number: 19-5-9 and in accordance with the Declaration of Helsinki and its later modifications.

### Study design

This study was conducted in the outpatient clinic of the Conservative Dentistry Department, Faculty of Dentistry at Cairo University. The study was a randomized controlled clinical trial with two parallel group designs with a 1:1 allocation ratio and superiority framework. The participants were randomly assigned into two groups (*n* = 15) according to the tested groups. Comprehensive periodontal and radiographic assessment for each case was performed at different time intervals, baseline, immediate postoperative, 6 months, and 12 months.

### Sample size calculation

This study aimed to assess the effectiveness of deep marginal acquisition using thermacut bur gingivectomy and deep marginal extension with direct composite restoration, versus functional crown lengthening in patients with deep sub-gingival interproximal carious lesions. Based on the previous research^8^ the response within each subject group was normally distributed with a standard deviation of 0.4. Using power 80% and 5% significance level we needed to study 11 subjects in each group. This number was increased to 15 to compensate for possible losses during follow-up. The sample size was calculated using PS power & sample version 3.1.6 for Windows using T-test.

### Eligibility criteria

Forty participants were recruited, and all posterior teeth were screened. Ten participants were excluded as they didn’t meet the inclusion criteria, and two others declined to participate to achieve a total of 30 eligible participants. The inclusion criteria of participants where male or female participants with age not less than 18 years old, having good oral hygiene, presence of at least one deep sub-gingival interproximal carious lesion. Eligible participants should have teeth with proximal margins violating the biological width with 1–2 mm distance from crestal bone level (CBL). teeth must be vital with no periapical radiolucency and don’t show any signs and symptoms of acute pulpitis. Eligible participants should have sufficient cognitive ability to understand consent procedures. The patients should be Co-operative and show interest in participating in the study.

While for exclusion criteria of participants were presence of disabilities, presence of systemic diseases or severe medically compromised (Cardiovascular disorder, diabetes, hypertension, epileptic, blood disorder). Patients who are allergic to any ingredients used in the study. Patients had participated in a clinical trial within 6 months before the commitment of this trial. Presence of severe or active periodontal disease. Patients had received therapeutic irradiation to the head and neck region. Patients who were unable to return for recall appointments.

### Recruitment

**Fig. 1:**
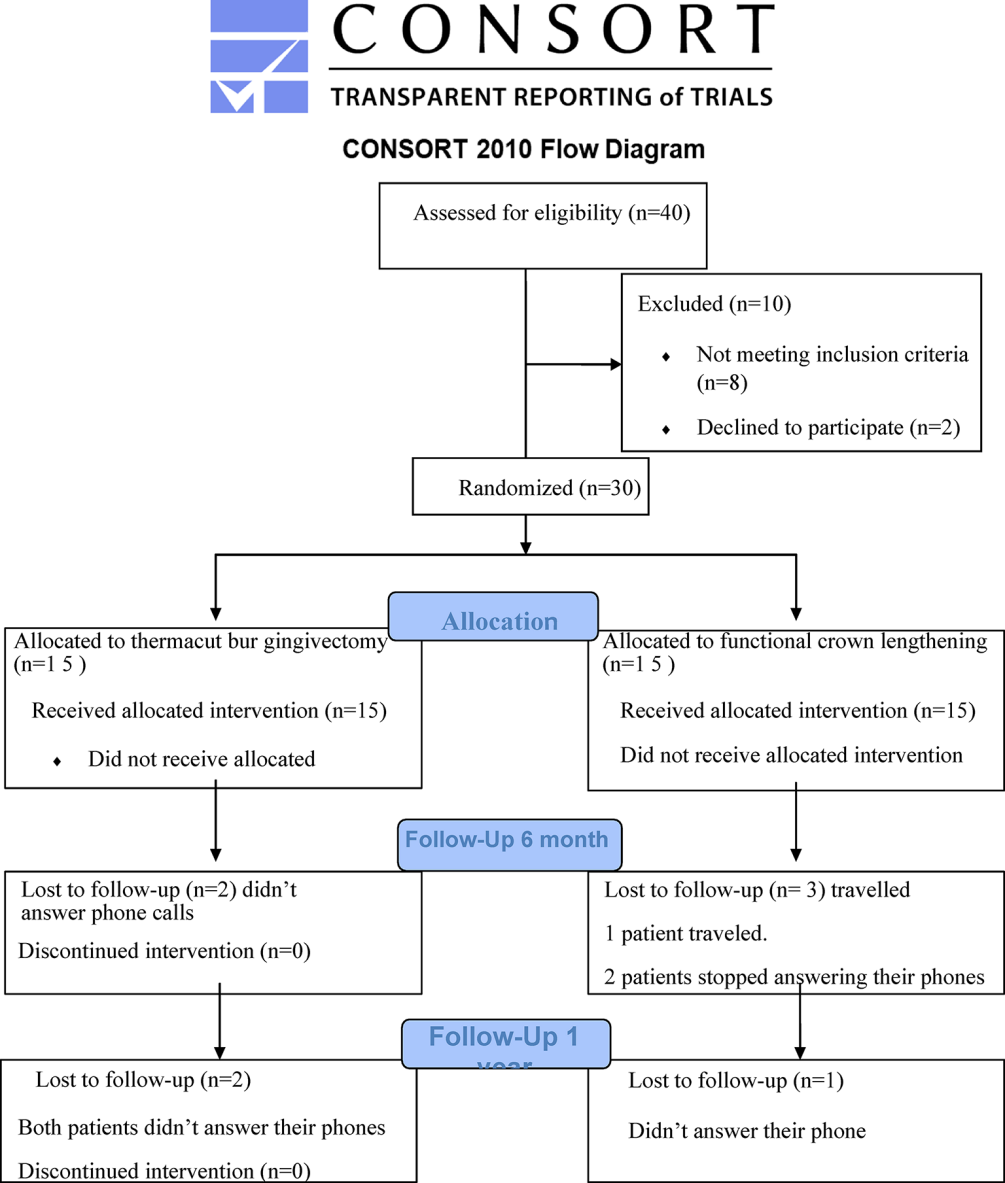
Flow chart of the study.

Patients were recruited for this clinical trial from the Conservative clinic, Faculty of Dentistry, Cairo University, where there is a continuous and high patient flow matching the inclusion criteria. Patients who fulfilled the eligibility criteria were recruited according to the participant timeline. For eligible participants, the primary investigator explained all aspects of the trial and was able to have an informed discussion about the procedures. Before enrollment, all participants signed a written informed consent after being completely aware of the settings of the study regarding the aim of performing the research, procedures expected to be done in detail including the number of visits, benefits for the participants, and possible side effects that might occur. All consent forms were written in Arabic to be well understood by all the participants. A consort flow diagram showing the flow of the participants through each stage of the current randomized clinical trial of the study. (Fig. 1)

### Randomization, allocation of participants and concealment

Simple randomization was done by generating numbers from 1:30 using Random Sequence Generator, Randomness and Integrity Services Ltd (https://www.random.org/) 2019 to reduce selection bias. The randomization list was kept securely away from the operator and the participants to ensure no tampering with the random list. Each participant has chosen a sequentially numbered opaque sealed envelope. Participants choose the envelope after signing the written informed consent. After the participant chose an envelope, it was signed by the patient. The number on the envelope was recorded in the patient chart to ensure the patient was assigned to the randomized group.

### Blinding

Neither the patients nor the operator was blinded due to the different nature of the two procedures, deep marginal acquisition using thermacut bur in the intervention group, and functional crown lengthening in the control group. Yet, two expert assessors were blinded to the assigned treatment of each patient, to reduce performance and detection bias. The statistician responsible for the processing of the result was blinded as well.

### Preoperative examination procedures

**Fig. 2:**
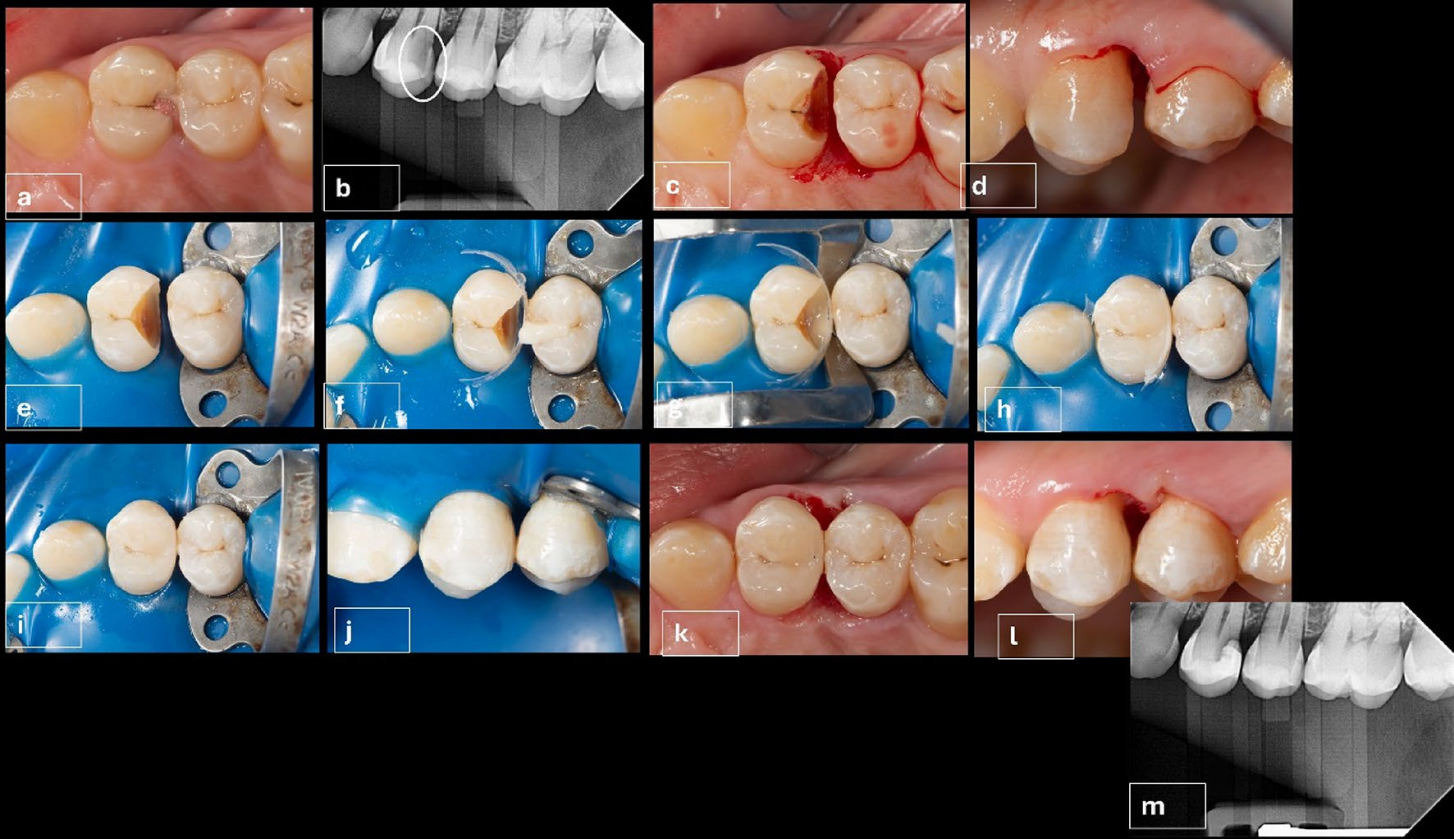
(**a**-**m**): Clinical procedures for the Intervention group (Thermacut bur); **a**: Preoperative photograph, **b**: Preoperative radiograph to measure alveolar bone level, **c**: Occlusal view after Papilla removed with thermacut bur, **d**: Buccal view for removed papilla to expose deep margin, **e**: Rubber dam placement and cavity preparation, **f**: Sectional matrix placement, **g**: Cervical hip build-up using bulk-fill flowable, followed by Elliot separator placement, **h**: Composite restoration before excess material removal, **i**: Removal of excess composite from buccal and palatal aspects, **j**: Buccal view showing emergence profile of the restoration, **k**: Immediate postoperative occlusal view showing restoration after finishing and polishing, **l**: Immediate postoperative buccal view showing restoration after finishing and polishing, **m**: Immediate postoperative radiograph to assess crestal bone level.

Patients with deep sub-gingival interproximal carious lesions were selected. Since carious lesions are associated with plaque deposits, the dental plaque must be removed before the assessment. Dental prophylaxis (scaling and polishing) was performed with a bristle brush and fluoridated prophylaxis paste, preoperative photograph, figure (2a), Preoperative pocket depth using a graded periodontal probe (Nordent manufacturing Inc. USA), and preoperative bitewing radiograph was taken for each participant. figure (2b).

### The intervention group (thermacut bur)

First, local anesthesia (4% articaine with 1:100000 epinephrine, ArtPharma, Egypt) was given to create field anesthesia using 1.4 ml, then the remaining 0.3 ml was administered to the papilla from both buccal and palatal or lingual aspects to reduce pain and bleeding during papilla removal(Venuti P. and Mirabella Eclano., 2018)^7^. Then a thermacut bur mounted on a high-speed 1:5 contra-angle handpiece (Joy Dental, China) was used to cut the papilla without coolant to acquire deep subgingival margin^7^, and to convert the location of the gingival seat from subgingival to a supragingival location. figures (2c, d).

The rubber dam was used to obtain isolation of the operative field. “6 × 6’’ heavy blue sanctuary rubber dam sheet (Sanctuary, Perak, Malaysia) was applied. The dam and frame were carried to the patient’s mouth first, then the clamp was placed to stabilize the dam at the tooth distal to the tooth to be restored. Then, interproximal placement of the rubber dam sheet and inversion of the dam margins into the gingival sulcus was achieved using dental floss. figure (2e).

Proximal box cavity preparation was performed in all cases by the mean of round diamond burs sizes (009,012, 021) (Komet Dental, Lemgo, Germany) mounted on a 1:5 contra-angle handpiece with 4-way water spray coolant (Joy Dental, China) at 200,000 RPM (revolution per minute). The size of the diamond bur was chosen based on carious lesion depth and extension to clean Enamel and Dentino-enamel junction (DEJ), accessing the proximal cavity from an occlusal direction through the triangular fossa. The cavity outline was driven by carious lesion extension, followed by sharp excavators (Dentsply, Konstanz, Germany), allowing for accessible removal of remaining soft carious lesions using scrapping motion instead of scooping action to avoid pulpal exposure. figure (2f).

Cleaning the cavity was done using Aquacare air abrasion device (Velopex, UK) by utilizing (29 μm) particle-sized aluminum oxide powder, followed by washing for 15 s to wash out the excess powder. A transparent contoured sectional matrix (Tor Vm, Russia) was placed. (Fig. 2f) The Elliot separator was placed afterward to adapt the matrix cervically and to compensate for the matrix thickness to attain contact tightness. figure (2 g).

Acid-etch gel was only applied to enamel (selective enamel etching technique) using a syringe, it was placed for 15–20 s, then rinsed using air and water stream for 20 s, then air dryness until the enamel had a chalky white appearance. After that, 2 coats of All-bond universal adhesive were applied to the enamel and dentin using a micro brush till completely wetting the surface. Vigorous rubbing and agitation were done for 20 s for each coat, followed by air plotting using a gentle stream of air until a uniform layer was formed. Then light curing for 20 s using Elipar S10 (3 m ESPE, USA) light curing unit.

In extremely deep cases, delayed wedging was performed by building up a 1.5 mm cervical hip using flowable Bulkfill composite before the Elliot separator was placed. It was done to prevent collapsing the matrix contour cervically or creating an under hang in the restoration, ensuring the matrix was properly self-adapted to the cavity margin and, in some cases, adaptation was enhanced using Teflon tape based on the clinical scenario. Figures (2 h).

The incremental composite placement was done starting by building up the proximal wall using a combination of flowable and packable composite resin (snow-plow technique). This was to ensure cavity margins were completely covered with composite and decrease the risk of void formation. The choice of Filtek one bulk-fill was to allow curing light to reach the deepest layers of the composite at the very deep margins, and to decrease polymerization shrinkage stresses. figure (2i, j).

The restorations were checked for adaptation to the cavity margins using an explorer, the excess material was removed using Eccesso instrument (LM-ARTE, Parainen, Finland), and the contour was adjusted to minimize the finishing step using yellow coded fine tapered with round end finishing stone (Mani, Tochigi, Japan). figure (2k, l) Polishing was done using Diacomp Twist polishing rubbers (EVE GmbH, Germany) mounted on a 1:1 low-speed contra-angle handpiece with internal coolant (Kavo, Germany) at 10,000 RPM. An explorer was used to ensure no excess material at the tooth-restorative interface and proper adaptation of the restoration, by moving it from the tooth to the restoration and moving it to the opposite way to ensure no open margins existed. Immediate postoperative bitewing radiograph was taken after completion of restoration to evaluate crestal bone level (CBL), figure (2 m), and pocket depth was measured using a graded periodontal probe.

### The preoperative examination procedure for the control group (surgical crown lengthening)

**Fig. 3:**
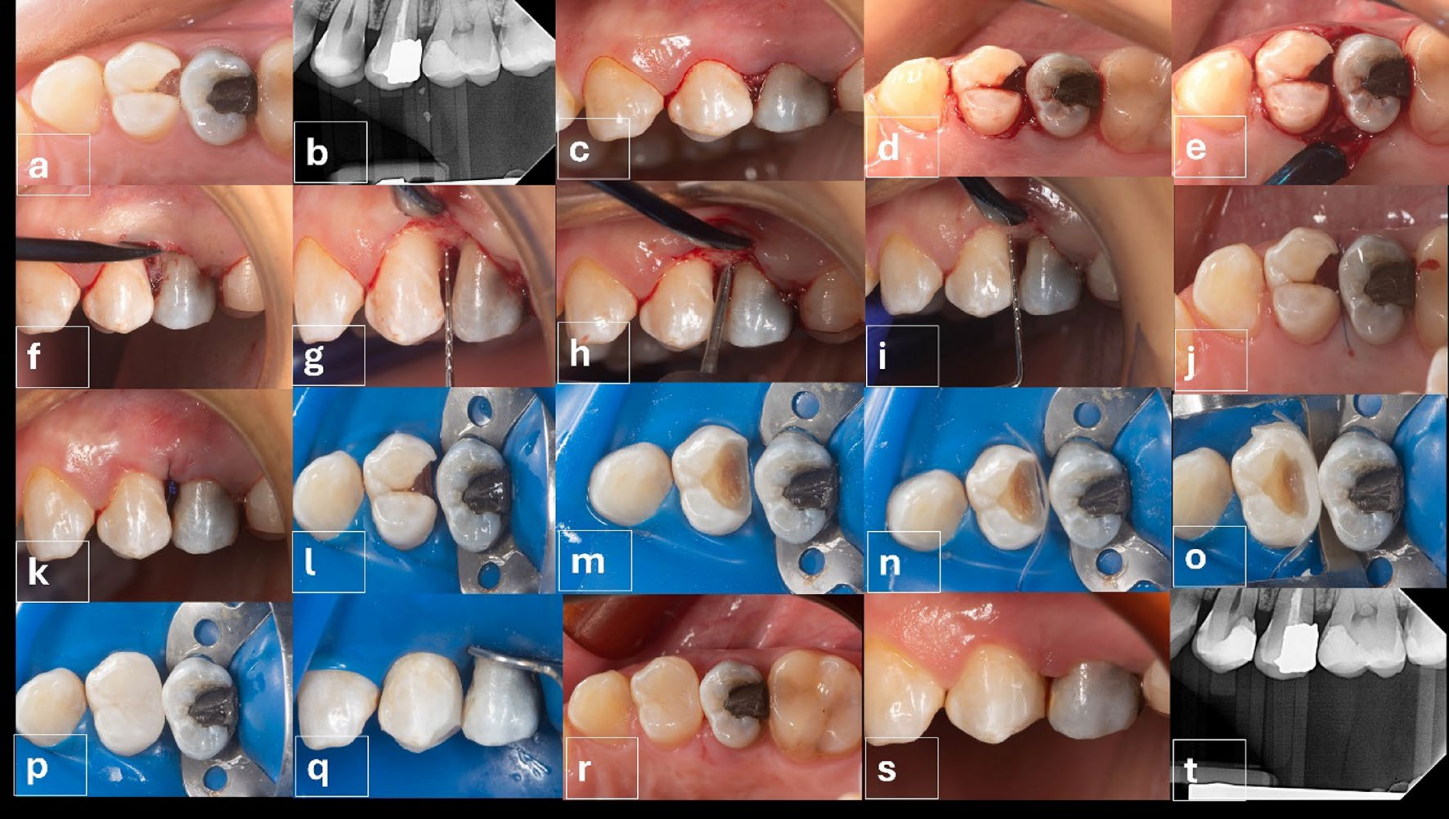
(**a**-**t**): Clinical procedures for the control group (Surgical crown lengthening), **a**: Preoperative occlusal view for the case representing control group, **b**: Preoperative radiograph representing the control group, **c**: Buccal view showing sulcular incision, **d**: Occlusal view showing sulcular incision, **e**: Palatal aspect of flap reflection, **f**: Buccal aspect of flap reflection, **g**: Measuring distance between the alveolar bone crest and cavity margin using a graded periodontal probe, **h**: Lowering bone level using End-cutting bur, **i**: Measuring distance between the alveolar bone crest and cavity margin using a graded periodontal probe after bone level modification, **j**: Occlusal view after suturing the flap, **k**: buccal view after suturing the flap, **l**: Rubber dam isolation, **m**: Cavity preparation and performing partial carious lesion removal, **n**: Sectional matrix placement, **o**: Proximal wall build-up, **p**: Composite restoration after finishing, **q**: Buccal view showing emergence Profile of composite restoration, **r**: 10 days postoperative occlusal view after suture removal, **s**: Buccal view of a 10 day follow up after suture removal, **t**: Immediate postoperative radiograph to evaluate bone level.

Similar preoperative patient preparation was carried out as previously described for plaque removal, dental prophylaxis, pocket depth determination and preoperative bitewing radiograph records. figures (3a,b)

### The surgical protocol for the control group

First, local anesthesia was given to create field anesthesia using 1.5 ml, then the remaining 0.3 ml for palatal or lingual injections to reduce pain during surgical procedures. The surgical procedures started with a sulcular incision using a 15c blade, extending from the mesiobuccal aspect to the distobuccal aspect of the tooth with an internal bevel incision at the tooth of interest. figures (3c, d) A full thickness mucoperiosteal minimal flap reflection with a small mucoperiosteal elevator, followed by flap raising to access the area to observe the bone tissue and the attached gingival fibers. figure (3e, f).

Root scaling and polishing were performed through Gracey curettes sizes #7–8, #11–12, and #13–14 (Hu-Friedy Mfg. Co., LLC, Chicago, IL, USA) around all teeth and roots exposed by total flap raising. Soft tissue collar was removed when needed and the crestal bone level was measured using a graded periodontal probe, figure (3 g), all flap extensions were aimed to verify which areas of the bone contour needed to be modified for crown lengthening.

Osteoplasty was performed using an end-cutting bur to avoid any injury to the root, (Fig. 2 h), with constant irrigation, and minimal contouring of the buccal bone crest to avoid having a reverse architecture in the bone level, followed by measuring 2.5–3 mm from the cavity margin till the bone crest to ensure having sufficient biological width, figure (3 h, i). The flap was sutured with 5/0 (Egyprolene, Egypt). An external vertical mattress suturing technique was used to ensure soft tissue adaptation to bone and cavity margin exposure. figures (3j, k).

### Restorative protocol for the control group

The same restorative procedures were done in both groups, to eliminate the confounding factor between the intervention and control group, by waiting for 6 weeks until healing occurred and temporization of the cavity, and based on the work ofTseng, Fu JH, and Wang HL, 2011^9^and Gomes Tortoriello et al., 2016^10^. It was decided to place the restoration at the same visit of the functional crown lengthening procedure. (figure a-t)

### Data collection, management, and analysis

The baseline data were collected for this clinical trial through diagnostic charts containing medical and dental histories. The process of data collection was rechecked to avoid any missing or incomplete data.

## Results

### Demographic data

The study was conducted on 22 cases (i.e., 11 cases per group). There were 4 males and 7 females in the intervention group with a mean age of (32.90 ± 8.01) years, while in the control group, there were 3 males and 8 females with a mean age of (34.10 ± 8.31) years. There was no significant difference between both groups regarding gender distribution and age (*p* > 0.05).

### Primary outcome: patient satisfaction using visual analog scale (VAS)

Inter, intragroup comparisons and summary statistics for (VAS) are presented in Table (2). Immediately postoperative, cases in the control group had significantly higher pain scores than those in the test group (*p* < 0.001). However, starting from 6 months, all cases in both groups had a zero score. Within both groups, there was a significant difference between values measured in different intervals, with values measured immediately postoperative being significantly higher than other intervals’ values (*p* < 0.001).

**Table 2:** Inter, intragroup comparisons and summary statistics for (VAS).

Time	Measurement	(VAS)		Test statistic	p-value
		Intervention (Thermacut bur gingivectomy)	Control (Functional crown lengthening)		
Immediately postoperative	Mean±SD	0.55±0.69 A	1.82±0.40 A	110.50	<0.001*
	Median (IQR)	0.00 (1.00)A	2.00 (0.00)A		
6 months	Mean±SD	0.00±0.00B	0.00±0.00B	NA	NA
	Median (IQR)	0.00 (0.00)B	0.00 (0.00)B		
1 year	Mean±SD	0.00±0.00B	0.00±0.00B	NA	NA
	Median (IQR)	0.00 (0.00)B	0.00 (0.00)B		
Test statistic		6.92	222.22		
p-value		0.005*	<0.001*		

NA: Not Applicable. Values with different superscripts within the same vertical column are significantly different *; significant (p < 0.05). .

VAS: Visual analogue scale. IQR: interquartile range.

### Secondary outcome

#### Bleeding on probing (BoP)

Inter, intragroup comparisons, and summary statistics for bleeding on probing status are presented in Table (3). Preoperatively and immediately postoperatively, all cases in both groups had bleeding on probing. After 6 months, 3 cases in the intervention group and 2 cases in the control group were positive and the difference was not statistically significant (*p* = 0.611). Similarly, after 1 year, 2 cases in the intervention and a single case in the control group were still positive and the difference was not significant (*p* = 0.534). Within both groups, there was a significant difference between values measured at different intervals, with the percentage of positive cases at 6 months and 1 year being significantly lower than those measured preoperatively and immediately postoperatively (*p* < 0.001).

**Table 3:** Inter, intragroup comparisons and summary statistics for bleeding on probing status.

**Time**	**Bleeding on probing**	**Bleeding on probing status [n (%)]**		**Test statistic**	**p-value**
		**Intervention** **(Thermacut bur gingivectomy)**	**Control** **(Functional crown lengthening)**		
**Preoperative**	**No**	0 (0.00%)^A^	0 (0.00%)^A^	**NA**	**NA**
	**Yes**	11 (100.00%)	11 (100.00%)		
**Immediately postoperative**	**No**	0 (0.00%)A	0 (0.00%)A	**NA**	**NA**
	**Yes**	11 (100.00%)	11 (100.00%)		
**6 months**	**No**	8 (72.73%)^B^	9 (81.82%)B	**0.26**	**0.611ns**
	**Yes**	3 (27.27%)	2 (18.18%)		
**1 year**	**No**	9 (81.82%)^B^	10 (90.91%)^B^	**0.39**	**0.534ns**
	**Yes**	2 (18.18%)	1 (9.09%)		
**Test statistic**		**24.94**	**27.92**		
**p-value**		<0.001*	<0.001*		

NA: Not Applicable. Values with different superscripts within the same vertical column are significantly different *; significant (p<0.05), ns; non-significant.

### Pocket depth (PD)

Inter, intragroup comparisons and summary statistics for (PD) are presented in Table (4). Preoperative and 6-month intervals, there was no significant difference in values measured in both groups (*p* > 0.05). In the immediate postoperative results, the control group had significantly higher values than the intervention (*p* < 0.001). However, after one year, values measured in the intervention group were significantly higher (*p* = 0.038). Within the intervention group, there was a significant difference between values measured in different intervals, with values measured immediately postoperative being significantly lower than those of other intervals (*p* < 0.001). While for the control group, the difference was not statistically significant (*p* = 0.106).

**Table 4:** Inter, intragroup comparisons and summary statistics for (PD).

Time	Measurement	(PD) (mm)		Test statistic	p-value
		Intervention (Thermacut bur gingivectomy)	Control (Functional crown lengthening)		
Preoperative	Mean±SD	2.82±0.25 A	2.68±0.25 A	77.00	0.226ns
	Median (IQR)	3.00 (0.50)A	2.50 (0.50)A		
Immediate postoperative	Mean±SD	0.18±0.25B	2.68±0.25 A	121.00	<0.001*
	Median (IQR)	0.00 (0.50)B	2.50 (0.50)A		
6 months	Mean±SD	2.77±0.26 A	2.59±0.20 A	82.50	0.091ns
	Median (IQR)	3.00 (0.50)A	2.50 (0.00)A		
1 year	Mean±SD	2.82±0.25 A	2.59±0.20 A	88.00	0.038*
	Median (IQR)	3.00 (0.50)A	2.50 (0.00)A		
Test statistic		415.79	2.22		
p-value		<0.001*	0.106ns		

Values with different superscripts within the same vertical column are significantly different *; significant (p < 0.05) ns; non-significant (p>0.05).

#### Crestal bone level evaluation (CBL)

Inter, intragroup comparisons and summary statistics for crestal bone level (mm) are presented in Table (5). Preoperatively, there was no significant difference in bone levels measured in both groups (*p* = 0.549). In contrast, in other intervals, values measured in the control group were significantly higher than the interventions’ (*p* < 0.001). Within both groups, there was a significant difference between values measured at different intervals (*p* < 0.001). For the intervention group, post hoc pairwise comparisons showed preoperative values to be significantly higher than values measured immediately postoperatively and after 6 months (*p* < 0.001). For the control group, they showed values measured after 6 months and 1 year to be significantly higher than other intervals’ values (*p* < 0.001). In addition, they showed values measured immediately postoperatively to be significantly higher than preoperative values (*p* < 0.001).

**Table 5:** Inter, intragroup comparisons and summary statistics for crestal bone level (mm).

Time	Measurement	Bone level (mm)		Test statistic	p-value
		Intervention (Thermacut bur gingivectomy)	Control (Functional crown lengthening)		
Preoperative	Mean±SD	1.92±0.32 A	1.96±0.09 C	70.00	0.549ns
	Median (IQR)	2.00 (0.60)A	2.00 (0.20)C		
Immediate postoperative	Mean±SD	1.76±0.30B	2.50±0.17B	121.00	<0.001*
	Median (IQR)	1.90 (0.50)B	2.50 (0.10)B		
6 months	Mean±SD	1.79±0.29B	2.60±0.17 A	121.00	<0.001*
	Median (IQR)	2.00 (0.40)B	2.60 (0.10)A		
1 year	Mean±SD	1.83±0.30 AB	2.62±0.15 A	121.00	<0.001*
	Median (IQR)	2.00 (0.40)AB	2.60 (0.10)A		
Test statistic		7.98	264.83		
p-value		<0.001*	<0.001*		

Values with different superscripts within the same vertical column are significantly different *; significant (p < 0.05).

### Associations between crestal bone level & bleeding on probing

Associations between Crestal Bone Level and Bleeding on Probing are presented in Table (6). For the intervention group, the association was not statistically significant (*p* = 0.486). For the control group, the association was statistically significant with bone level in cases free from bleeding being significantly higher than that of affected cases (*p* < 0.05).

**Table 6:** Associations between Crestal bone level and bleeding on probing.

**Group**	**Measurement**	**Bone level (mm)**		**Test statistic**	**p-value**
		**BOP (no)**	**BOP (yes)**		
**Intervention** **(Thermacut bur gingivectomy)**	**Mean±SD**	1.87±0.23	1.80±0.33	**258.00**	**0.486ns**
	**Median (IQR)**	2.00 (0.40)	1.90 (0.50)		
**Control** **(Functional crown lengthening)**	**Mean±SD**	2.64±0.15	2.22±0.33	**430.00**	<0.001*

*;significant (p<0.05), ns; not significant.

### Tertiary outcome: evaluating marginal integrity using modified USPHS criteria

**Fig. 4:**
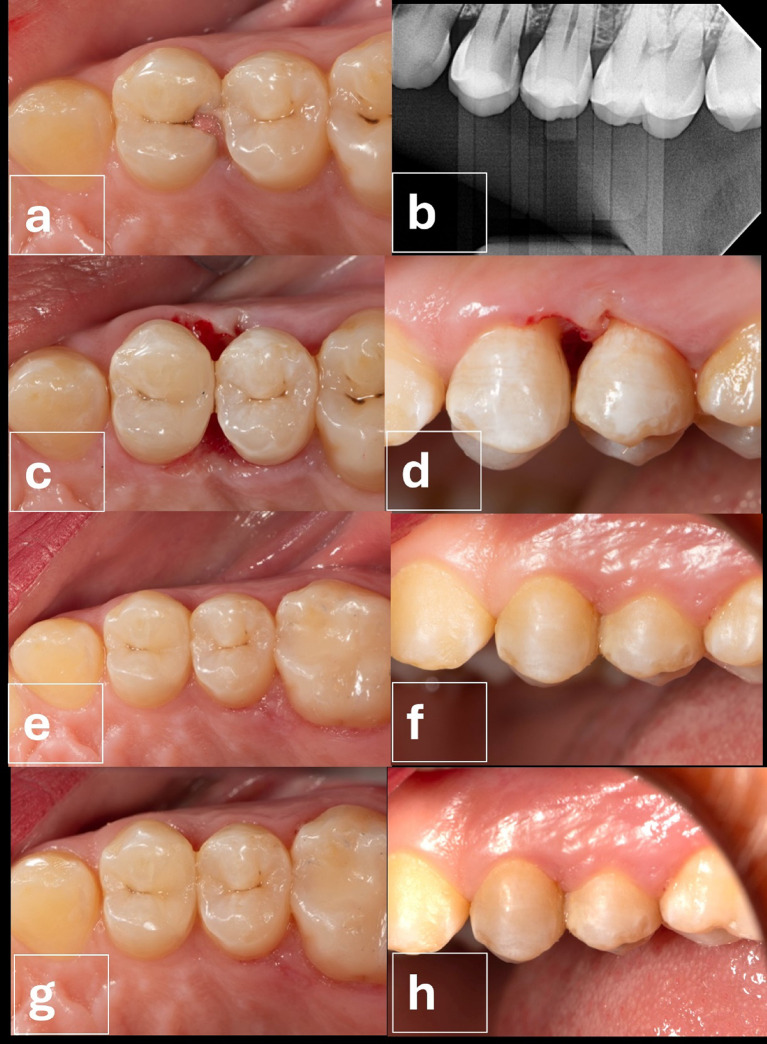
(**a**-**h**): Follow up intervention group; **a**: Pre- occlusal view, **b**: preoperative radiograph, **c**: immediate post- occlusal view, **d**: Immediate postoperative buccal view, **e**: 6-month follow-up occlusal view, **f**: 6- month follow-up buccal view, **g**: 1-year follow-up occlusal view, **h**: 1 year follow-up buccal view.

**Fig. 5:**
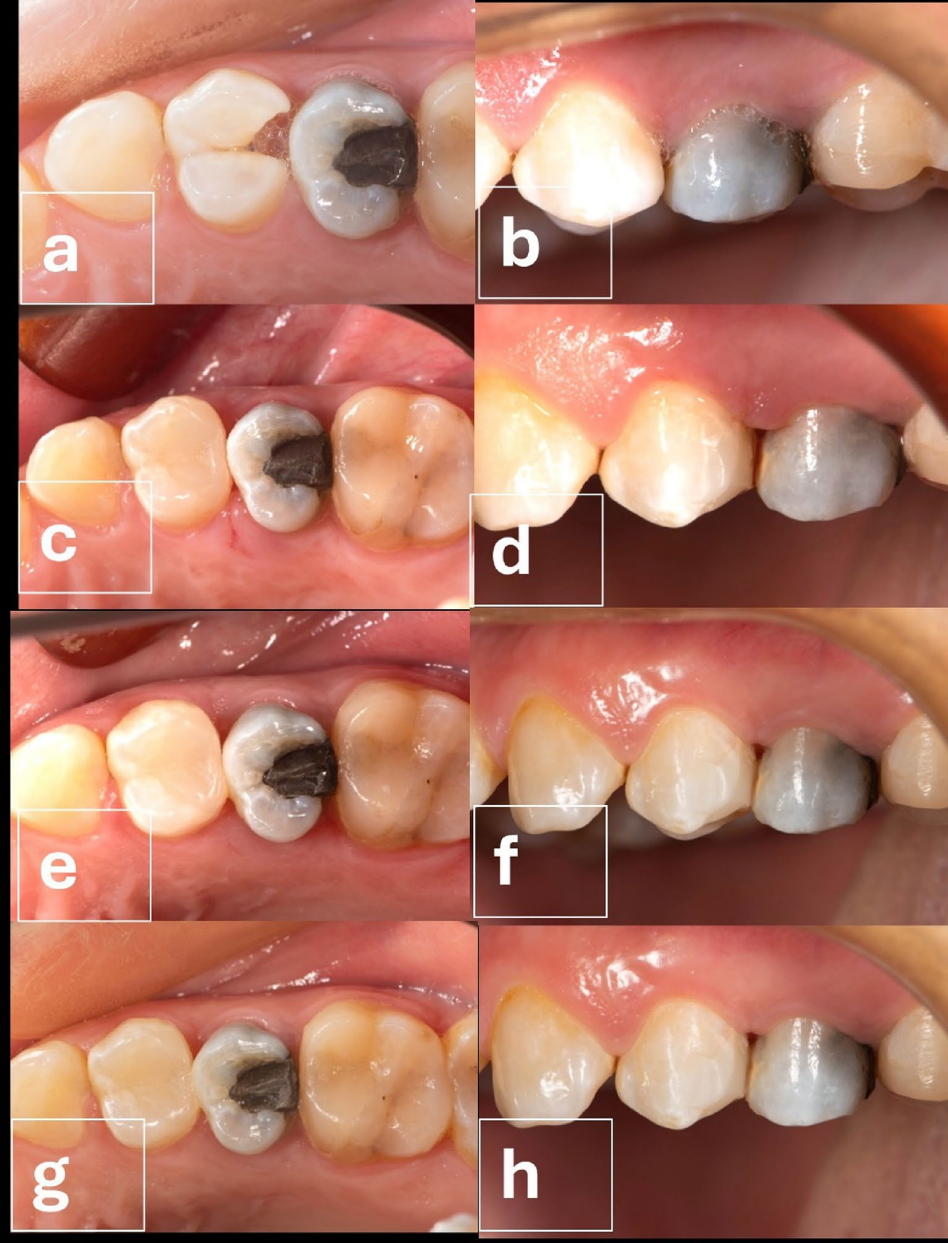
(**a**-**h**): Follow up Control group Surgical crown lengthening; **a**: Preoperative occlusal view, **b**: Preoperative buccal view, **c**: 10-day follow-up occlusal view after suture removal, **d**: 10-day follow-up buccal view after suture removal, **e**: 6-month follow-up occlusal view, **f**: 6-month follow-up buccal view, **g**: 1-year follow-up occlusal view, **h**: 1-year follow-up buccal view.

All cases in both groups had an alpha score during all follow-up intervals regarding marginal staining (Table 7) and marginal adaptation (Table 8). However, for surface roughness, at baseline, all cases in both groups had an alpha score. Starting from 6 months, two cases in the intervention group had a bravo score, yet the difference between both groups was not statistically significant (*p* = 0.167). (Table 9; Figs. 4 and 5)

**Table 7:** Inter, intragroup comparisons and summary statistics for Marginal Staining scores.

**Time**	**Score**	**Marginal integrity scores [n (%)]**		**Test statistic**	**p-value**
		**Intervention** **(Thermacut bur gingivectomy)**	**Control** **(Functional crown lengthening)**		
**Preoperative**	**Alpha**	11 (100.00%)	11 (100.00%)	**NA**	**NA**
	**Bravio**	0 (0.00%)	0 (0.00%)		
	**Charlie**	0 (0.00%)	0 (0.00%)		
**6 months**	**Alpha**	11 (100.00%)	11 (100.00%)	**NA**	**NA**
	**Bravio**	0 (0.00%)	0 (0.00%)		
	**Charlie**	0 (0.00%)	0 (0.00%)		
**1 year**	**Alpha**	11 (100.00%)	11 (100.00%)	**NA**	**NA**
	**Bravio**	0 (0.00%)	0 (0.00%)		
	**Charlie**	0 (0.00%)	0 (0.00%)		
**Test statistic**		**NA**	**NA**		
**p-value**		**NA**	**NA**		

NA: Not Applicable.

**Table 8:** Inter, intragroup comparisons and summary statistics for Marginal Adaptation scores.

**Time**	**Score**	**Marginal adaptation scores [n (%)]**		**Test statistic**	**p-value**
		**Intervention** **(Thermacut bur gingivectomy)**	**Control** **(Functional crown lengthening)**		
**Baseline**	**Alpha**	11 (100.00%)	11 (100.00%)	**NA**	**NA**
	**Bravo**	0 (0.00%)	0 (0.00%)		
	**Charlie**	0 (0.00%)	0 (0.00%)		
**6 months**	**Alpha**	11 (100.00%)	11 (100.00%)	**NA**	**NA**
	**Bravo**	0 (0.00%)	0 (0.00%)		
	**Charlie**	0 (0.00%)	0 (0.00%)		
**1 year**	**Alpha**	11 (100.00%)	11 (100.00%)	**NA**	**NA**
	**Bravo**	0 (0.00%)	0 (0.00%)		
	**Charlie**	0 (0.00%)	0 (0.00%)		
**Test statistic**		**NA**	**NA**		
**p-value**		**NA**	**NA**		

NA: Not Applicable.

**Table 9:** Inter, intragroup comparisons and summary statistics for Surface Roughness scores.

**Time**	**Score**	**Surface roughness scores [n (%)]**		**Test statistic**	**p-value**
		**Intervention** **(Thermacut bur gingivectomy)**	**Control** **(Functional crown lengthening)**		
**Baseline**	**Alpha**	11 (100.00%)	11 (100.00%)	**NA**	**NA**
	**Bravo**	0 (0.00%)	0 (0.00%)		
	**Charlie**	0 (0.00%)	0 (0.00%)		
**6 months**	**Alpha**	9 (81.82%)	11 (100.00%)	**71.50**	**0.167ns**
	**Bravo**	2 (18.18%)	0 (0.00%)		
	**Charlie**	0 (0.00%)	0 (0.00%)		
**1 year**	**Alpha**	9 (81.82%)	11 (100.00%)	**71.50**	**0.167ns**
	**Bravo**	2 (18.18%)	0 (0.00%)		
	**Charlie**	0 (0.00%)	0 (0.00%)		
**Test statistic**		**4.00**	**NA**		
**p-value**		**0.135ns**	**NA**		

NA: Not Applicable, ns; non-significant (p>0.05).

## Discussion

Deep subgingival interproximal carious lesions can seriously affect direct restorative workflow, complicating cavity preparation, isolation, matrix placement, separation, and adhesion procedure of the direct restoration. Three decades ago, (Dietschi D and Spreafico R., 1998)^6^introduced Cervical Margin Relocation which was renamed by (Magne and Spreafico, 2012)^2^into Deep Margin Elevation. However, this technique was intended for cavity design optimization (CDO) by modifying the remaining tooth structure to receive indirect restoration, the decision was to use direct composite restoration rather than an indirect approach in the current study^11^.

In the current study, Patient Satisfactionthroughevaluating overall experience including pain and discomfort was the primary outcome measurement. It was chosen because it is a patient-related outcome, representing an important aspect that is usually overlooked in the literature^12^. Patient-related outcomes are of great importance in clinical practice, where input from the patients has a great value in measuring their overall experience. A Visual Analog Scale (VAS) was used to assess the level of pain.

For the primary outcome, there was a statistically significant difference in patient satisfaction in both intra- and inter-group comparisons in the immediate postoperative results, showing higher values in the control group. This may be attributed to the effect of the surgical procedure from flap reflection, root scaling, bone removal, and suturing which may increase the level of pain. This was compared to papilla removal using a thermacut bur, and only after anesthesia was injected into the papilla before removal. It is worth mentioning that in one specific case in the intervention group, the pain score was 2, however, the pain wasn’t from papilla removal. Instead, it was from discomfort related to mouth opening, caused by rubber dam^13^.

These results came into agreement with the work of Antoniazzi et al., 2014^14^and Farouk et al., 2024^15^. who found that FCL caused more pain in the VAS compared to deep marginal elevation. They attributed the cause of pain to flap raising, bone removal, and suturing. Furthermore, in the current study, no statistically significant difference was found in the 6-month and one year intervals in both intra- and inter-group comparisons. It is plausible during these follow-up periods that healing has occurred, pain subsided, and patient satisfaction increased, which was confirmed by the clinical and radiographic observations.

Secondary outcomes including Bleeding on Probing (BoP), Pocket Depth (PD), and Crestal Bone level (CBL) were measured using a graded periodontal probe and bitewing radiograph with a paralleling technique, respectively. As for BoP, all cases in both groups had bleeding on probing during the preoperative evaluation which can be explained by the presence of cavitation and food impaction, which caused inflammation and subsequent bleeding on probing.

However, after 6 months, only 3 cases in the intervention group and 2 cases in the control group had bleeding on probing. Also, after 1 year, 2 cases in the intervention and a single case in the control group had BoP, and the differences weren’t statistically significant in both time intervals. Within both groups, there was a significant decrease in BoP in the follow-up intervals. These results showed a favorable response of the periodontium to both presented treatment modalities. It is also worth mentioning that maintaining good oral hygiene was paramount to achieving these results.

These results came into agreement with the work of Oppermann et al., 2016^16^and Farouk et al., 2024^15^, showing a significant decrease in BoP values after 6 months of follow-up and one year respectively. However, there was a disagreement with Ferrari et al., 2018^17^, who published a conflicting result concerning BoP, showing an increase in the incidence of BoP after 1-year follow-up. It can be explained by the presence of surface roughness of the flowable composite used for deep marginal elevation. The presence of overhanging in the flowable composite due to the inability to control the flowable composite placement using a matrix, or failure to remove excess composite material or adhesive flashes which might have persisted causing plaque accumulation, and interfered with oral hygiene measures, which can lead to a higher risk of bleeding on probing.

In the current clinical trial, all restorations were performed under magnification to ensure superior restorative procedures were being delivered to the patients, also patients were instructed to follow strict oral hygiene measures including tooth brushing and flossing to maintain a healthy periodontium. As for pocket depth, there was no statistically significant difference between preoperative and 6-month follow-up in both intra- and inter-group comparisons. However, there was a statistically significant difference between immediate postoperative measurement and different intervals within the intervention group, with the immediate postoperative measurement having the lowest value. This is due to the removal of the papilla with thermacut bur, which leads to the elimination of the sulcus causing the readings with graded periodontal probe near zero in all cases of intervention, at the immediate postoperative interval.

While; 1-year measurement was statistically significant in the intervention group, which might be attributed to either subclinical inflammation of the gingival margin neighboring the restoration making the reading slightly higher. It is worth mentioning that the increase in pocket depth within the intervention group wasn’t clinically significant enough to cause any patient-related complications, like pain during eating or clinically obvious signs of inflammation, including redness or bleeding on brushing. This also concurs with the work of Frese, Wolff and Staehle, 2014^18^, Venuti P. and Mirabella Eclano., 2018^7^, andFarouk et al., 2024^15^ in which there was neither clinical significance in pocket depth, nor patient-related complications.

Within the control group, there was no statistically significant difference between pocket depth at different time intervals. Which indicated clinical reattachment and healing after the functional crown lengthening. These clinical findings could be attributed to many reasons as described by Pontoriero and Carnevale, 2001^19^. The result of a remodeling process that occurs in the periodontal tissues over 6 & 12 months post-surgical procedures, is that a new supra-crestal gingival unit was created. These results also concur with the work of Arora et al., 2013^20^, in which Crown length gained during surgery significantly decreased 6 months post-surgery, indicating tissue rebound and healing.

A very interesting clinical insight that should be mentioned here, which may explain the statistically significant difference in PD between the two groups is the tissue rebound. Which remarkably favored the intervention group with no apparent black triangles beneath contact areas under restorations, while; in the control group, some cases showed small black triangles below contact points. The reason for that can be due to the reduction of crestal bone height, which support the papilla and the modification of the alveolar bone crest architecture.

In the intervention group, and because of the preservation of the alveolar bone crest position and architecture, the papilla healed and closed the gap fully under the contact areas. However, in the control group, the alveolar bone crest was intentionally modified to preserve the papilla and prevent the infringement of the so-called biological width. This caused modification of the positive architecture of the alveolar bone crest, and increased the distance between the apical part of the contact area and the alveolar bone crest which might cause the presence of small black triangles under the contact area and in turn making the PD values appear less. However, as mentioned before it wasn’t a clinically significant^21^.

The biocompatibility of resin composite materials with the surrounding periodontal tissue depends on many criteria. It includes the chemistry of the polymerizable organic matrix of the resinous material, degree of conversion, type of ceramic fillers, and degree of conversion of the material^22^. Resin composite materials used for the direct restorative procedure were bulk-fill nano-based materials, which are characterized by reduced free monomers due to a higher degree of conversion^23,24^. These features resulted in better biocompatibility and better periodontal tissue response.

Another explanation for such results both in intervention and control groups can be explained by proper composite restoration placement and curing against the matrix surfaces, which were highly surface polished. Surface smoothness plays an important role in decreasing biofilm attachment to the restoration leading to better healing of the soft tissue around composite restorations. This concurs with the work of Frese, Wolff, and Staehle, 2014^18^and Samartzi et al., 2022^25^. Their clinical observations showed that plain, smooth, and non-irritating margins on deep interproximal composite restorations infringing the junctional epithelium could be free of gingival and periodontal inflammation. It should be noted that strict oral hygiene measures were followed.

Regarding **crestal bone level** measurements, there was a statistically significant difference between both groups at all time intervals, which can be explained by the difference in nature of both procedures, as bone removal was only limited to the control group. For the intervention group, preoperative values were higher than immediate and in 6-month and 1-year intervals, which can be explained by the effect of cavity preparation causing the increase in depth of the cavity cervical margin, because of carious lesion removal.

However, no statistically significance difference occurred after 6-month and 1-year intervals, this can be a result of no disturbance of alveolar bone crest level. This came into agreement with the work of Frese, Wolff, and Staehle, 2014^18^and Farouk et al., 2024^15^, which showed very minimal changes to crestal bone level over 12 months. This indicated that polished, smooth, and nonirritating subgingival margins can prevent any negative impact on periodontal tissues.

For the control group, 6-month and 1-year intervals showed statistically significant differences when compared to immediate postoperative values, which might be plausible due to the healing which -according to literature- can take up to 6 months. It concurs with the work of Pontoriero and Carnevale, 2001^19^and Farouk et al., 2024^15^, which showed that during 1 year of healing after functional crown lengthening, the periodontal tissue rebounded and grew in a coronal direction from the level defined at surgery, which was more pronounced in patients with thick tissue biotypes and also appeared to be influenced by individual variations in the healing response, not related to age or gender.

Concerning the association between Crestal Bone Level and Bleeding on Probing, no statistical significance within the intervention group was found. Thermacut bur gingivectomy is a novel technique, and more studies; including histological studies may be needed to explain the occurrence of such results. While for the control group, the association was statistically significant with bone level in cases free from bleeding being significantly higher than that of affected cases. It can be interpreted by the occurrence of a non-destructive inflammatory response toward the treatment modality of the control group.

An interesting point to be considered is that based on the work of Schmidt et al., 2013^3^, biological width dimensions undergo variations even in the same patient and after FCL was done. It can explain why BoP was associated more when CBL had fewer values. As mentioned above. BoP can be affected by a variety of reasons such as surface roughness, overhanging, and underhanging of restorations. In the current study, no negative clinical outcomes were reported regarding BoP or CBL as a response to both clinical modalities.

As for Tertiary Outcome which is restoration evaluation based on Modified USPHS Criteria, assessment of Marginal Integrity including **marginal staining**,** marginal adaptation**,** and surface discoloration**as outcome measurements, as for marginal adaptation, there were no statistically significant differences between both groups in both intra- and inter-group comparisons at different time intervals. This can be explained by the good quality of the restorative procedure that had been provided, considering all the procedural steps during composite placement to ensure optimal final results. It can also emphasize the need for a longer follow-up to verify the results^26^.

For surface roughness, there was no statistically significant difference between both groups in both intra- and inter-group comparisons at different time intervals. However, only 2 cases showed a “Bravo” score which was further investigated. The reason was the type of toothbrush when both patients mentioned the use of a medium toothbrush, which may explain the reason for the surface discoloration score being “Bravo”. However, there was neither a statistically nor clinically significant difference^26^. (Celik et al., 2010)

A report was recently published in January 2025 by the same team, as a pilot study featuring same parameters of the current study. The results of the 22 months follow-up case report were very promising in the terms of restorative and periodontal aspects. Which laid the foundation for treatment for the intervention group. (Elmorsy et al., 2025)

Another clinical insight can be added to this discussion, no secondary carious lesions were found in any case while reporting for follow-up sessions. Concurring with the work of Frese, Wolff, and Staehle, 2014^18^, Venuti P. and Mirabella Eclano., 2018^7^and Farouk et al., 2024^15^. It indicated a good quality of the restorative treatment was done according to the procedural steps during composite placement to ensure an optimal outcome. It also emphasized the need for a longer-term follow-up to verify these results.

Thus, based on the clinical findings of this clinical trial, the null hypothesis can be partially accepted. There was no clinically significant difference across the measurement criteria between thermacut bur gingivectomy and functional crown lengthening. The main differences were in patient satisfaction and only in the immediate postoperative time interval, as it favored the thermacut bur gingivectomy over the functional crown lengthening technique due to the reasons mentioned above, and the Crestal bone level which increased as a result of the inherent procedural difference between both techniques.

### Limitations of the study

Since the study involved molars and premolars, it will be challenging to create a dry accessible field for restorative procedures. The authors emphasize on the use of rubber dam to properly control moisture. Tooth position, either upper or lower, or type of tooth (molar, premolar) or accessibility of the tooth, all these challenges should be taken into consideration for each treatment procedure. It was strongly recommended to properly polish, clean, air-abrade, and dryness of the tooth surface. Moreover, chronic interproximal carious lesions may limit the accessibility of the thermacut bur between the teeth to remove the papilla due to continuous mesial drifting of teeth. Caries extension subgingivally, compromised crown root ratio, deep cavities beside implant crowns, risking furcation involvement, and risking black triangle formation below the restoration must be considered in treatment plan. All these confounders were considered and controlled in this study and were overcome in proper case selection via the properly designed eligibility criteria to eliminate any bias during the research.

The authors of the study highlight the importance of using magnification to properly assess marginal acquisition and isolating deep margins, either by thermacut bur of functional crown lengthening. Also the authors highly recommend extreme caution during papilla removal at lower second molar region to avoid cutting through buccal mucosa and causing tissue emphysema.

## Conclusions

Within the limitations of this study the following conclusions can be drawn:

- Thermacut bur gingivectomy can be recommended because of reduced immediate postoperative pain, length of the procedure, and no need for extra specialty involvement in the treatment of deep interproximal carious lesions.
- Thermacut bur can be considered superior to functional crown lengthening in terms of preserving the alveolar bone crest.

## Recommendations

- Thermacut bur gingivectomy is an alternative to solve the shortcomings of functional crown lengthening and it is highly recommended in cases with compromised crown root ratio, deep cavities beside implant crowns, risking furcation involvement, and risking black triangle formation below the restoration.
- Studies with longer-term follow-up may be needed to verify the findings of this study related to the durability of the direct esthetic restoration.
- Histological studies might be needed to explain the negative correlation between BoP and CBL when thermacut bur was used.

## Clinical significance

- Thermacut bur gingivectomy can be introduced as an easy technique for clinicians to manage deep subgingival interproximal carious lesions, without the need to refer patients unnecessarily to periodontists and without the need for special devices.
- Elliot separator is a great tool for matrix adaptation and creating an optimum separation potential.

### Statistical analysis

Categorical data were presented as frequency and percentage values and were analyzed using Fisher’s exact test. Numerical data were presented as mean, standard deviation (SD), median, and interquartile range (IQR) values. They were analyzed for normality by viewing the data distribution and using Shapiro-Wilk’s test. Age data were normally distributed and were analyzed using an independent t-test. Other data were non-parametric and were analyzed using the Mann-Whitney U test for intergroup comparisons and Friedman’s test, followed by Nemenyi’s post hoc test for intragroup comparisons. The significance level was set at *p*< 0.05 within all tests. Statistical analysis was performed with R statistical analysis software version 4.3.3 for Windows^27^.

## Electronic supplementary material

Below is the link to the electronic supplementary material.

Supplementary Material 1

## Data Availability

All data generated are included in the current manuscript and available upon reasonable request to the corresponding author.

## Abbreviations

MDP: 10-methacryloyloxydecyl dihydrogen phosphate
HEMA: Hydroxy ethyl methacrylate
UDMA: Urethane dimethacrylate resin
TEGDMA: Triethyleneglycol dimethacrylate
AUDMA: Aliphatic urethane dimethacrylate resin
DMA: Dimethacrylate resins
TBG: Thermacut bur gingivectomy
DME: Deep margin elevation
FCL: Functional crown lengthening
BoP: Bleeding on probing
PD: Probing depth
CBL: Crestal bone level
CDO: Cavity design optimization
mm: millimeters
PBE: Proximal box elevation
CMR: Cervical margin relocation
VAS: Visual analog scale
CBL: Crestal bone level
