# Supplementary material for: Thermacut bur gingivectomy versus functional crown lengthening in management of subgingival interproximal carious lesions 12 months randomized controlled trial
Source: Sci Rep. 2025 May 8;15:16063. doi: 10.1038/s41598-025-99313-0 (PMC12062263; doi:10.1038/s41598-025-99313-0)
Supplement: Supplementary file 1 — Supplementary Material 1 [file 41598_2025_99313_MOESM1_ESM.docx]

**Supplementary table (1):** Tertiary outcome measurements

| Clinical Characteristics | Marginal Adaptation | Surface roughness | Marginal staining |
| --- | --- | --- | --- |
| Alpha | Explorer does not catch or has one-way catch when drawn across the restoration/tooth interface. | The surface of the restoration does not have any surface defects | There is no discoloration between the restoration and tooth. |
| Bravo | Explorer does not catch or has one-way falls into crevice when drawn across the restoration/tooth interface. | The surface of the restoration has minimal surface defects | There is discoloration on less than half of the circumferential margin. |
| Charlie | Dentin or base is exposed along the margin. | The surface of the restoration has severe surface defects | There is discoloration on more than half of the circumferential margin. |
| Delta | Debonding of the restoration | N/A | N/A |
